# Fasting triglycerides are positively associated with cardiovascular mortality risk in people with diabetes

**DOI:** 10.1093/cvr/cvac124

**Published:** 2022-07-29

**Authors:** Yutang Wang, Yan Fang, Dianna J Magliano, Fadi J Charchar, Christopher G Sobey, Grant R Drummond, Jonathan Golledge

**Affiliations:** Discipline of Life Science, School of Science, Psychology and Sport, Federation University Australia, University Drive, Mt Helen, Ballarat, VIC, 3350, Australia; Discipline of Life Science, School of Science, Psychology and Sport, Federation University Australia, University Drive, Mt Helen, Ballarat, VIC, 3350, Australia; Diabetes and Population Health, Baker Heart and Diabetes Institute, Melbourne, VIC, Australia; Discipline of Life Science, School of Science, Psychology and Sport, Federation University Australia, University Drive, Mt Helen, Ballarat, VIC, 3350, Australia; Centre for Cardiovascular Biology and Disease Research and Department of Microbiology, Anatomy, Physiology and Pharmacology, School of Agriculture, Biomedicine and Environment, La Trobe University, Melbourne, VIC, Australia; Centre for Cardiovascular Biology and Disease Research and Department of Microbiology, Anatomy, Physiology and Pharmacology, School of Agriculture, Biomedicine and Environment, La Trobe University, Melbourne, VIC, Australia; Queensland Research Centre for Peripheral Vascular Disease, College of Medicine and Dentistry, James Cook University, Townsville, QLD, Australia; Department of Vascular and Endovascular Surgery, The Townsville University Hospital, Townsville, QLD, Australia

**Keywords:** Hypertriglyceridaemia, Mortality, CVD, Diabetes, Risk factor

## Abstract

**Aims:**

We investigated the association of fasting triglycerides with cardiovascular disease (CVD) mortality.

**Methods and results:**

This cohort study included US adults from the National Health and Nutrition Examination Surveys from 1988 to 2014. CVD mortality outcomes were ascertained by linkage to the National Death Index records. Cox proportional hazards models were used to estimate hazard ratios (HRs) and 95% confidence intervals (CIs) of triglycerides for CVD mortality. The cohort included 26 570 adult participants, among which 3978 had diabetes. People with higher triglycerides had a higher prevalence of diabetes at baseline. The cohort was followed up for a mean of 12.0 years with 1492 CVD deaths recorded. A 1-natural-log-unit higher triglyceride was associated with a 30% higher multivariate-adjusted risk of CVD mortality in participants with diabetes (HR, 1.30; 95% CI, 1.08–1.56) but not in those without diabetes (HR, 0.95; 95% CI, 0.83–1.07). In participants with diabetes, people with high triglycerides (200–499 mg/dL) had a 44% (HR, 1.44; 95% CI, 1.12–1.85) higher multivariate-adjusted risk of CVD mortality compared with those with normal triglycerides (<150 mg/dL). The findings remained significant when diabetes was defined by fasting glucose levels alone, or after further adjustment for the use of lipid-lowering medications, or after the exclusion of those who took lipid-lowering medications.

**Conclusion:**

This study demonstrates that fasting triglycerides of ≥200 mg/dL are associated with an increased risk of CVD mortality in patients with diabetes but not in those without diabetes. Future clinical trials of new treatments to lower triglycerides should focus on patients with diabetes.

**Time of primary review: 41 days**

**Translational perspective**

There has been much interest in investigating whether lowering triglyceride levels protects against cardiovascular disease (CVD). However, 12 of 13 randomized controlled trials since 2010 have not demonstrated any effect of lowering triglycerides on CVD events and mortality. Our study found that elevated triglycerides were associated with enhanced CVD mortality in those with diabetes, but not in those without diabetes. The results of our study may offer some guidance for future clinical trials investigating the effect of lowering triglycerides on CVD: both diabetes and hypertriglyceridaemia may need to be used as inclusion criteria.

## 1. Introduction

Cardiovascular disease (CVD) is the leading global cause of mortality and a major contributor to disability.^1^ CVD prevalence nearly doubled from 271 million in 1990 to 523 million in 2019, and the number of CVD deaths increased from 12.1 million in 1990 to 18.6 million in 2019.^1^ Therefore, it is of high importance to identify modifiable risk factors for CVD and to decrease CVD mortality.

There has been much interest in investigating whether lowering triglyceride levels protects against CVD. However, 12 of 13 randomized controlled trials since 2010^2–14^ (see Supplementary material online, *Table S1*) have not demonstrated any effect of lowering triglycerides (via omega-3 fatty acid, niacin, or fibrate) on CVD events and mortality, challenging the belief that lowering triglycerides lowers CVD risk.

Recent reports suggest that triglycerides may be important for the pathogenesis of diabetes, a disease that can increase CVD risk. For example, higher baseline triglycerides were associated with higher risk of new-onset of diabetes^15–17^ and diabetes-caused mortality^18^ in cohort studies.

The bezafibrate infarction prevention (BIP) trial showed that in patients with established coronary heart disease, high baseline triglycerides predicted high all-cause mortality after adjustment for baseline diabetes diagnosis.^19^ However, whether there is an interaction between diabetes and triglycerides in predicting CVD mortality is unknown.

This cohort study aimed to investigate the association of fasting triglycerides with CVD mortality in US adult participants with or without diabetes who attended the National Health and Nutrition Examination Surveys (NHANES) from 1988 to 2014.

## 2. Methods

### 2.1 Study participants

This cohort study included participants from NHANES III (1988–1994) and the subsequent eight cycles of NHANES from 1999 to 2014.^20,21^ The inclusion criteria included age of ≥20 years and presence of fasting triglyceride data, resulting in a cohort of 27 184 people. The following were excluded: those who were pregnant (*n* = 582), those without a follow-up time or with a follow-up time of 0 month (*n* = 30), and those without diabetes status (*n* = 2). Therefore, 26 570 participants were included in the final analysis.

The National Centre for Health Statistics Research Ethics Review Board approved all study protocols.^18,20,22^ All procedures were performed following the guidelines of the Declaration of Helsinki. Written informed consent was obtained from all participants.

### 2.2 Diabetes definition

Diabetes was defined as presence of any of the following: self-reported physician diagnosis of diabetes, use of insulin or oral diabetes medications, haemoglobin A_1c_ (HbA_1c_) ≥6.5%, or fasting glucose ≥140 mg/dL (≥7.8 mmol/L) in NHANES III (1988–1994) or ≥126 mg/dL (≥7.0 mmol/L) in NHANES 1999–2014.^23^ NHANES was not conducted between 1995 and 1998. The use of different fasting plasma glucose levels for diabetes diagnosis was due to the change in diagnostic criteria over time, and the fasting glucose level for diabetes was ≥140 mg/dL before the American Diabetes Association criteria in 1997.^24^

### 2.3 Fasting triglycerides classification

The baseline concentration of fasting (fasting time ≥8 h^23,25^) triglycerides in the serum was directly retrieved from the NHANES website.^18^ Triglyceride levels were classified into four groups according to the recommendation by the National Cholesterol Education Program (NCEP) Expert Panel,^26^ i.e. normal (<150 mg/dL), borderline high (150–199 mg/dL), high (200–499 mg/dL), and very high (≥500 mg/dL).

### 2.4 CVD mortality

Data on mortality were directly retrieved from NHANES-linked mortality files.^18,20,22^ To evaluate mortality status, the National Centre for Health Statistics conducted probabilistic matching to link the NHANES data with death certificate records from the National Death Index (NDI) records. CVD mortality was defined as mortality from heart diseases or cerebrovascular diseases, as previously reported.^20^ Follow-up time was defined as the time (in months) from when the blood was drawn at the Mobile Examination Centre until death, or until the end of follow-up (i.e. 31 December 2015), whichever occurred first.^18,20,22^

### 2.5 Covariates

Confounding covariates were similar to previous reports.^18,20,22,27^ They included age (continuous), sex (male or female), ethnicity (Hispanic, non-Hispanic white, non-Hispanic black, or other),^28^ obesity (underweight, normal, overweight, obese, or unknown), education (<high school, high school, >high school, or unknown), poverty–income ratio (<130%, 130–349%, ≥ 350%, or unknown), and survey periods (1988–1991, 1991–1994, 1999–2000, 2001–2002, 2003–2004, 2005–2006, 2007–2008, 2009–2010, 2011–2012, or 2013–2014). Lifestyle confounders included physical activity (inactive, insufficiently active, or active), alcohol consumption (never, <1 drink per week, 1–6 drinks per week, ≥7 drinks per week, or unknown), and smoking status (past smoker, current smoker, non-smoker, or unknown). Clinical confounders included self-reported physician diagnosis of hypertension (yes, no, or unknown), self-reported physician diagnosis of hypercholesterolaemia (yes, no, or unknown), diabetes (yes or no), family history of diabetes (yes, no, or unknown), duration of diabetes (≥10 years, <10 years, or unknown), and diabetes medications (insulin only, oral medications only, both insulin and oral medications, or unknown).

### 2.6 Statistical analyses

Statistical analysis methods were similar to previous reports.^18,20,22^ Data were presented as mean and standard deviation for normally distributed continuous variables or median and interquartile range for non-normally continuous distributed variables or percentages for categorical variables. Difference in age was analysed using Student’s *t*-test between those with or without diabetes or one-way analysis of variance (ANOVA) among four triglyceride groups. Differences in non-normally distributed continuous variables (triglyceride, glucose, and HbA_1c_) were analysed using the Mann–Whitney *U* test between those with or without diabetes or using Kruskal–Wallis one-way ANOVA among four triglyceride groups. Differences among categorical variables were analysed using Pearson’s χ^2^ test. Cox proportional hazards models were used to calculate hazard ratios (HRs) and 95% confidence intervals (CIs) of triglycerides for CVD mortality, with adjustment for age, sex, ethnicity, obesity, poverty–income ratio, education, physical activity, alcohol consumption, smoking status, survey period, hypercholesterolaemia, hypertension, diabetes, family history of diabetes, duration of diabetes, and diabetes medications. Triglyceride was treated as a continuous variable (natural log-transformed) or a categorical variable (normal, borderline high, high, and very high).^26^ Subgroup analyses were conducted in those with or without pre-existing CVD which was defined as prior diagnosis of myocardial infarction or stroke, or in those with various levels of low-density lipoprotein cholesterol (≤55, 55.1–70, 70.1–100, and >100 mg/dL).^29,30^

Sensitivity analyses were conducted by defining diabetes according to fasting plasma glucose alone, or by further adjustment for the use of lipid-lowering medications, or by exclusion of those who took lipid-lowering medications. Sensitivity analyses were also conducted by adjusting for total cholesterol (continuous), high-density lipoprotein (HDL) cholesterol (continuous), or non-HDL cholesterol (continuous) instead of hypercholesterolaemia,^31^ or by adjusting for systolic blood pressure (continuous) instead of hypertension status.

The restricted cubic spline model (with five knots at 5th, 27.5th, 50th 72.5th, and 95th percentiles)^32^ was used to examine the shape of the association between triglycerides and CVD mortality in participants with or without diabetes, with adjustment for age, sex, ethnicity, obesity, poverty–income ratio, education, physical activity, alcohol consumption, smoking status, survey period, hypercholesterolaemia, hypertension, family history of diabetes, duration of diabetes, and diabetes medications.

The null hypothesis was rejected with a two-tailed *P*-value of <0.05. Restricted cubic spline analyses were performed using SAS® OnDemand for Academics (SAS Institute Inc, Cary, NC, USA) and all other analyses were performed using SPSS version 27.0 (IBM SPSS Statistics for Windows; IBM Corporation, Armonk, NY, USA).

## 3. Results

### 3.1 General characteristics

This study included 26 570 adult participants, among whom 3978 had diabetes. The baseline characteristics of the participants are displayed in *Tables 1 and 2.* People with higher triglycerides had a higher prevalence of diabetes, and people with diabetes had higher triglycerides compared with those without diabetes. People with diabetes (compared with those without diabetes), as well as those with higher triglycerides, were more likely to be males, had less income and education, and had a higher prevalence of obesity, hypercholesterolaemia, and hypertension (*Tables 1 and 2*).

**Table 1:** Baseline characteristics of 26 570 US adults stratified by diabetes status

	Participants without diabetes	Participants with diabetes	All participants	*P-*value
Sample size	22 592	3978	26 570	NA
Age, years, mean (SD)	47 (18)	61 (14)	49 (19)	<0.001
Sex (female), %	51.3	49.6	51.1	0.047
Ethnicity, %				<0.001
ȃHispanic	27.1	30.8	27.7	
ȃNon-Hispanic white	46.1	39.6	45.1	
ȃNon-Hispanic black	21.7	24.0	22.1	
ȃOther	5.1	5.5	5.1	
Triglyceride, mg/dL, median (IQR)	105 (74–153)	142 (98–209)	109 (77–161)	<0.001
FPG, mg/dL, median (IQR)	96 (89–102)	133 (114–169)	97 (90–107)	<0.001
HbA_1c_, %, median (IQR)	5.3 (5.1–5.6)	6.7 (6.0–7.8)	5.4 (5.1–5.8)	<0.001
Obesity, %				
ȃUnderweight	1.8	0.7	1.7	<0.001
ȃNormal	34.8	15.1	31.9	
ȃOverweight	34.6	31.9	34.2	
ȃObese	27.8	50.2	31.2	
ȃUnknown	0.9	2.1	1.1	
Poverty–income ratio, %				
ȃ<130%	27.8	33.3	28.6	<0.001
ȃ130–349%	36.9	37.9	37.0	
ȃ ≥ 350%	27.1	19.3	26.0	
ȃUnknown	8.2	9.4	8.4	
Education, %				
ȃ<High school	30.5	43.0	32.4	<0.001
ȃHigh school	26.0	24.1	25.7	
ȃ>High school	43.2	32.7	41.7	
ȃUnknown	0.3	0.2	0.3	
Physical activity, %				
ȃInactive	28.0	18.5	26.6	<0.001
ȃInsufficiently active	38.2	29.3	36.9	
ȃActive	33.7	52.2	36.5	
Alcohol consumption, %				
ȃ0 drink/week	16.0	27.3	17.7	<0.001
ȃ<1 drink/week	22.5	20.8	22.2	
ȃ1–6 drinks/week	21.8	11.6	20.3	
ȃ ≥ 7 drinks/week	13.7	9.6	13.1	
ȃUnknown	26.0	30.7	26.7	
Smoking status, %				
ȃPast smoker	23.8	17.4	22.9	<0.001
ȃCurrent smoker	23.5	34.2	25.1	
ȃNon-smoker	52.6	48.3	51.9	
Hypercholesterolaemia, %	22.8	45.2	26.2	<0.001
Hypertension, %	26.9	60.2	31.9	<0.001
Diabetes, %	0	100	15.0	NA
Family history of diabetes, %	40.5	60.1	43.4	<0.001
Diabetes duration ≥10 years, %	0.0	22.2	3.3	NA
Use of diabetes medications, %				
ȃInsulin only	0.0	5.9	0.9	NA
ȃOral medications only	0.0	38.8	5.8	
ȃBoth	0.0	5.6	0.8	

HbA_1c_, haemoglobin A_1c_; FPG, fasting plasma glucose; IQR, interquartile range; NA, not applicable; SD, standard deviation.

**Table 2:** Baseline characteristics of 26 570 US adults stratified by triglyceride categories

	Triglyceride (mg/dL)				*P-*value
	<150	150–199	200–499	≥500	
Sample size	18 802	3714	3726	328	NA
Age, years, mean (SD)	47 (19)	53 (18)	53 (17)	49 (15)	<0.001
Sex (female), %	53.3	48.7	44.3	28.7	<0.001
Ethnicity, %					
ȃHispanic	43.1	49.3	50.8	47.9	<0.001
ȃNon-Hispanic white	26.3	13.5	10.5	8.2	
ȃNon-Hispanic black	25.2	32.5	34.4	39.6	
ȃOther	5.4	4.8	4.3	4.3	
Triglyceride, mg/dL, median (IQR)	89 (68–114)	170 (159–183)	251 (220–305)	642 (551–834)	<0.001
FPG, mg/dL, median (IQR)	96 (89–104)	101 (94–111)	103 (95–118)	110 (98–173)	<0.001
HbA_1c_, %, median (IQR)	5.4 (5.1–5.7)	5.5 (5.2–5.9)	5.6 (5.2–6.1)	5.6 (5.2–7.4)	<0.001
Obesity, %					
ȃUnderweight	2.1	0.6	0.5	0.3	
ȃNormal	37.6	20.7	15.9	11.0	<0.001
ȃOverweight	32.4	37.9	38.5	45.1	
ȃObese	26.8	39.7	43.9	43.0	
ȃUnknown	1.0	1.2	1.2	0.6	
Poverty–income ratio, %					
ȃ<130%	27.9	28.9	31.1	33.8	<0.001
ȃ130–349%	37.2	36.8	36.3	39.9	
ȃ≥350%	26.7	24.6	24.1	19.2	
ȃUnknown	8.2	9.7	8.5	7.0	
Education, %					
ȃ<High school	29.9	37.9	38.4	39.6	<0.001
ȃHigh school	25.6	25.6	26.3	25.9	
ȃ>High school	44.2	36.1	35.1	34.5	
ȃUnknown	0.3	0.3	0.2	0.0	
Physical activity, %					
ȃInactive	27.8	24.1	22.9	27.1	<0.001
ȃInsufficiently active	37.1	35.5	37.4	34.5	
ȃActive	35.0	40.4	39.7	38.4	
Alcohol consumption, %					
ȃ0 drink/week	17.0	19.2	19.8	18.9	<0.001
ȃ<1 drink/week	22.6	22.5	20.6	18.0	
ȃ1–6 drinks/week	21.3	17.7	18.2	19.8	
ȃ≥7 drinks/week	12.9	13.2	13.5	18.9	
ȃUnknown	26.3	27.4	27.9	24.4	
Smoking status, %					
ȃPast smoker	22.1	24.1	24.9	28.4	<0.001
ȃCurrent smoker	23.2	29.4	30.3	29.0	
ȃNon-smoker	54.6	46.4	44.7	42.7	
Hypercholesterolaemia, %	21.8	33.6	39.0	48.2	<0.001
Hypertension, %	28.7	37.6	41.1	41.8	<0.001
Diabetes, %	11.4	19.8	25.8	40.9	<0.001
Family history of diabetes, %	41.8	45.5	48.7	53.7	<0.001
Diabetes duration ≥10 years, %	2.7	4.0	5.2	7.9	<0.001
Use of diabetes medications, %					
ȃInsulin only	0.8	0.9	1.5	1.5	<0.001
ȃOral medications only	4.4	7.6	10.3	15.9	
ȃBoth	0.7	0.9	1.4	2.7	

HbA_1c_, haemoglobin A_1c_; FPG, fasting plasma glucose; IQR, interquartile range; NA, not applicable; SD, standard deviation.

### 3.2 Association of fasting plasma triglycerides with CVD mortality

This cohort was followed up for a mean of 12.0 years with a total of 318 346 person-years of follow-up. During the follow-up, 1492 CVD deaths were recorded.

A 1-natural-log-unit increase in triglycerides was not associated with CVD mortality in the whole cohort nor the non-diabetic subcohort (*Table 3*). However, it was associated with a 30% higher multivariate-adjusted risk of CVD mortality in participants with diabetes (HR, 1.30; 95% CI, 1.08–1.56; *Table 3*). Restricted cubic spline analyses showed that the association between triglycerides and CVD mortality risks in participants with diabetes was not linear (*P* = 0.011, Supplementary material online, *Figure S1*). When triglycerides were treated as a categorical variable, similar results were obtained, and people with high triglycerides (200–499 mg/dL) had a 44% higher multivariate-adjusted risk of CVD mortality compared with those with normal triglycerides (<150 mg/dL) in the subcohort of participants with diabetes (*Table 4*). Interaction analyses confirmed that diabetes status interacted with triglycerides for CVD mortality risks (*P* = 0.015, Supplementary material online, *Table S2*).

**Table 3:** Triglyceride (natural log-transformed) and risk for CVD mortality among 26 570 adults

Models	HR	95% CI	*P*-value
Overall (*n* = 26 570)			
ȃModel 1	1.24	1.12–1.36	<0.001
ȃModel 2	1.16	1.05–1.28	0.005
ȃModel 3	1.12	1.01–1.24	0.033
ȃModel 4	1.06	0.95–1.17	0.315
Participants without diabetes (*n* = 22 592)			
ȃModel 1	1.05	0.93–1.18	0.463
ȃModel 2	0.97	0.86–1.10	0.641
ȃModel 3	0.94	0.83–1.07	0.359
ȃModel 4	0.95	0.83–1.07	0.382
Participants with diabetes (*n* = 3978)			
ȃModel 1	1.41	1.18–1.68	<0.001
ȃModel 2	1.31	1.09–1.57	0.004
ȃModel 3	1.28	1.06–1.53	0.009
ȃModel 4	1.30	1.08–1.56	0.006

CI, confidence interval; CVD, cardiovascular disease; HR, hazard ratio.

Model 1: adjusted for age, sex, and ethnicity. Model 2: adjusted for age, sex, ethnicity, obesity, poverty–income ratio, education, physical activity, alcohol consumption, smoking status, and survey period. Model 3: adjusted for all the factors in Model 2 plus hypercholesterolaemia and hypertension. Model 4: adjusted for all the factors in Model 3 plus diabetes, family history of diabetes, duration of diabetes, and diabetes medications.

**Table 4:** Triglyceride categories and risk for CVD mortality among 26 570 adults

Triglyceride (mg/dL)	*n*	HR^a^	95% CI	*P*-value^b^	*P* for trend
All participants (*n* = 26 570)					
ȃ<150 (normal)	18 802	1	Reference	NA	0.668
ȃ150–199 (borderline high)	3714	1.02	0.88–1.17	0.830	
ȃ200–499 (high)	3726	1.04	0.91–1.20	0.557	
ȃ≥500 (very high)	328	1.25	0.86–1.83	0.243	
Participants without diabetes (*n* = 22 592)					
ȃ<150 (normal)	16 656	1	Reference	NA	0.575
ȃ150–199 (borderline high)	2978	0.97	0.83–1.14	0.718	
ȃ200–499 (high)	2764	0.88	0.74–1.05	0.160	
ȃ≥500 (very high)	194	0.93	0.48–1.81	0.840	
Participants with diabetes (*n* = 3978)					
ȃ<150 (normal)	2146	1	Reference	NA	0.015
ȃ150–199 (borderline high)	736	1.10	0.83–1.46	0.496	
ȃ200–499 (high)	962	1.44	1.12–1.85	0.004	
ȃ≥500 (very high)	134	1.66	1.01–2.70	0.044	

CI, confidence interval; CVD, cardiovascular disease; HR, hazard ratio.

Adjusted for age, sex, ethnicity, obesity, poverty–income ratio, education, physical activity, alcohol consumption, smoking status, survey period, hypercholesterolaemia, hypertension, diabetes, family history of diabetes, duration of diabetes, and diabetes medications.

Compared with those with normal triglycerides.

Subanalyses showed that triglycerides were positively associated with CVD mortality in participants with diabetes, regardless of pre-existing CVD status (*Table 5*). In addition, the positive association between triglycerides and CVD mortality in people with diabetes was only presented in those with low-density lipoprotein cholesterol concentrations ranging from 70.1 to 100 mg/dL (see Supplementary material online, *Table S3*).

**Table 5:** Triglyceride (natural log-transformed) and risk for CVD mortality among 26 413^a^ adults, stratified by diabetes and pre-existing CVD^b^

Groups	*n*	HR^c^	95% CI	*P*-value
Participants without diabetes				
ȃWithout pre-existing CVD	21 283	0.88	0.76–1.02	0.088
ȃWith pre-existing CVD	1179	1.01	0.77–1.31	0.967
Participants with diabetes				
ȃWithout pre-existing CVD	3309	1.29	1.02–1.63	0.032
ȃWith pre-existing CVD	642	1.54	1.09–2.18	0.015

CI, confidence interval; CVD, cardiovascular disease; HR, hazard ratio.

A total of 157 participants were excluded due to unknown status of pre-existing CVD. Therefore, the remaining 26 413 participants were included in the analysis.

Pre-existing CVD was defined as prior diagnosis of myocardial infarction or stroke.

Adjusted for age, sex, ethnicity, obesity, poverty–income ratio, education, physical activity, alcohol consumption, smoking status, survey period, hypercholesterolaemia, hypertension, family history of diabetes, duration of diabetes, and diabetes medications.

### 3.3 Sensitivity analyses

Sensitivity analyses showed that the association of triglycerides with CVD mortality did not materially change after diabetes was re-defined according to fasting glucose alone (see Supplementary material online, *Tables S4 and S5*), or after further adjustment for the use of lipid-lowering medications (see Supplementary material online, *Table S6*), or after exclusion of those who took lipid-lowering medications (see Supplementary material online, *Table S7*), or after adjusting for systolic blood pressure instead of hypertension status (see Supplementary material online, *Table S8*). In addition, adjustment for total cholesterol, HDL cholesterol, or non-HDL cholesterol instead of hypercholesterolaemia did not abolish the association between triglycerides and CVD mortality in people with diabetes (see Supplementary material online, *Tables S9–S11*).

## 4. Discussion

This study found that elevated triglycerides were associated with enhanced CVD mortality in those with diabetes, but not in those without diabetes, in a large cohort of US adults. The positive association between triglycerides and CVD mortality in people with diabetes was independent of prior diagnosis of CVD.

In epidemiological studies, diabetes has often been defined by self-reported physician diagnosis and use of diabetes medications. However, using self-reported diagnosis to identify diabetes could be inaccurate,^33^ and in a similar manner diabetes medications and HbA_1c_.^34^ Therefore, sensitivity analyses were conducted by defining diabetes using era-specific fasting plasma glucose alone or using the single fasting plasma glucose level of ≥126 mg/dL. As lipid-lowering medications could affect triglyceride levels,^35^ sensitivity analyses were also conducted by further adjustment for the use of those medications or by exclusion of those who took those medications. These sensitivity analyses did not materially affect the results. In addition, family history of diabetes, diabetes duration, and diabetes medications were adjusted for in all the analyses. Therefore, this study supports the conclusion that elevated fasting triglycerides were associated with increased possibility of CVD mortality in people with diabetes.

The current study was observational in nature and therefore could not establish whether elevated triglycerides are merely a marker of risk or a causative factor. Mendelian randomization studies showed that genetically higher triglycerides were associated with increased CVD risk,^36^ suggesting that elevated triglycerides are pathogenic and thus a potential therapeutic target.

**Figure 1:**
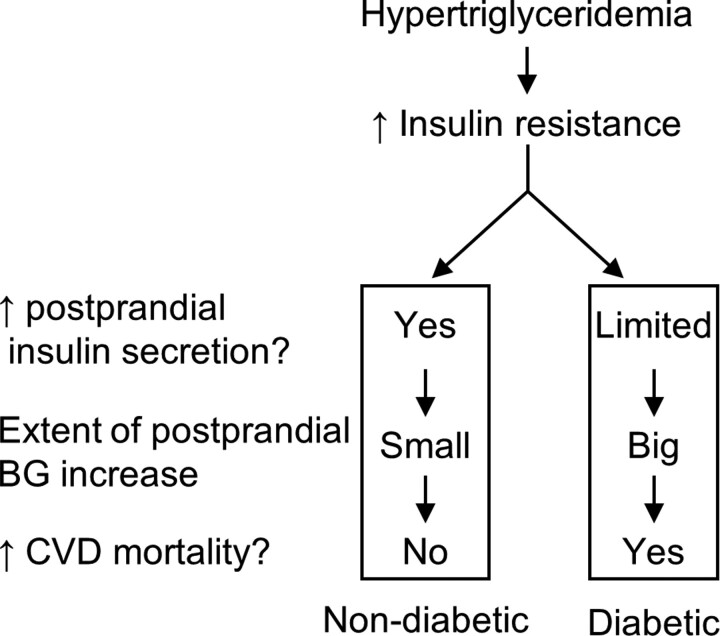
The proposed hypothesis of diabetes-induced sensitization to hypertriglyceridaemia-associated CVD mortality. High triglycerides induce insulin resistance. In people without diabetes, increased insulin resistance could be compensated by higher insulin secretion to maintain postprandial glucose homeostasis. However, in people with diabetes, the compensation capacity is limited, leading to a greater increase in postprandial BG, and ultimately, enhanced CVD mortality. ↑, increase; BG, blood glucose; CVD, cardiovascular disease.

This study indicates that hypertriglyceridaemia was associated with CVD mortality preferentially in people with diabetes as opposed to those without diabetes. The reason for this is not clear. The authors propose the following hypothesis for diabetes-induced sensitization to hypertriglyceridaemia (*Figure 1*).

Triglyceride-induced insulin resistance and impaired insulin secretion in diabetic patients might explain the proposed hypothesis. Infusion of triglycerides into rats induced insulin resistance as assessed by the euglycaemic-hyperinsulinaemic clamp study.^37^ Consistently, triglycerides in humans were positively associated with insulin resistance, the latter being assessed by homeostatic model assessment for insulin resistance.^18^ In people without diabetes, increased insulin resistance associated with high triglycerides could be compensated by higher insulin secretion to maintain postprandial glucose homeostasis. However, the insulin secretion in people with diabetes is impaired,^38^ and therefore, increased insulin resistance associated with high triglycerides could not be sufficiently compensated by an increase in insulin secretion. Consequently, high triglycerides could lead to a much higher plasma glucose increase after a meal in people with diabetes than in those without the disease. This may be supported by the following observation: triglycerides were linearly associated with plasma glucose in both non-diabetic and diabetic adults after adjustment for multiple confounders; however, the standardized coefficient (*β*) was 0.074 in non-diabetic adults, whereas it was 0.292 in diabetic adults.^18^ Plasma glucose is positively associated with CVD mortality^25^; consequently, the detrimental effect of triglycerides could be sensitized by diabetes.

The proposed diabetes-induced sensitization to hypertriglyceridaemia hypothesis is consistent with previous reports that higher baseline triglycerides were associated with new-onset of diabetes in various populations including Americans,^15^ Japanese,^16^ and Chinese,^17^ as well as with diabetes-caused mortality in people without diabetes at baseline.^18^ About 55% of patients with Type 2 diabetes have a triglyceride level higher than normal (i.e. >150 mg/dL),^39^ and this might, at least in part, explain why people with diabetes have a higher CVD risk (about two-fold on average) compared with those without the disease.^40,41^

The proposed diabetes-induced sensitization to hypertriglyceridaemia hypothesis is supported by the ACCORD-Lipid study.^14^ That study showed that although lowering triglycerides by fenofibrate did not decrease CVD risk in the overall cohort of patients with Type 2 diabetes,^14^ the therapy showed a 31% lower CVD event rate in the subcohort of diabetic patients with a combination of hypertriglyceridaemia and low HDL cholesterol.^14^ Our hypothesis is also supported by a study that showed that higher triglycerides are associated with CVD mortality in patients with diabetes,^42^ although, unlike the current study, that study^42^ had a small sample size (562 patients) and only 15 CVD deaths recorded.

Findings from the Baltimore Coronary Observational Long-Term Study (COLTS) appear to not support our proposed hypothesis, as higher triglycerides remained a significant risk factor for new CVD events after exclusion of Type 2 diabetes after 18 years of follow-up.^43^ However, the COLTS study finding may not reject the hypothesis, as during the 18-year follow-up, higher triglycerides may have led to diabetes formation in some participants, as higher triglycerides were associated with new-onset of diabetes^15–17^ and diabetes-caused mortaltiy^18^ in people without diabetes.

In addition, the 22-year mortality data from the BIP trial showed that, in patients with established coronary heart disease, high baseline triglycerides were associated with high all-cause mortality independent of baseline diabetes diagnosis,^19^ which may argue against such a diabetes-sensitization hypothesis. However, whether there was an interaction between diabetes and triglycerides in the association between triglycerides and all-cause mortality in that cohort^19^ was not investigated.

The findings of the current study suggest that people who may benefit most from triglyceride-lowering therapies are those with both diabetes and hypertriglyceridaemia. This study might provide a new explanation for why the majority of recently completed randomized controlled trials failed to demonstrate that lowering triglycerides protects against CVD, i.e. none of these trials used both diabetes and hypertriglyceridaemia as inclusion criteria (see Supplementary material online, *Table S1^2^*). Among these 13 trials, only three used hypertriglyceridaemia as an inclusion criterion (see Supplementary material online, *Table S1^2^*): Reduction of Cardiovascular Events with Icosapent Ethyl–Intervention Trial (REDUCE-IT),^9^ Long-Term Outcomes Study to Assess Statin Residual Risk with Epanova in High Cardiovascular Risk Patients with Hypertriglyceridaemia (STRENGTH),^10^ and Atherothrombosis Intervention in Metabolic Syndrome with Low HDL/High Triglycerides, Impact on Global Health Outcomes (AIM-HIGH) trials.^12^ The AIM-High trial had a low percentage of participants with diabetes (33.9%),^12^ whereas the REDUCE-IT^9^ and STRENGTH^10^ trials had a higher prevalence of diabetes (58.5 and 70%, respectively; see Supplementary material online, *Table S1^2^*). Hypertriglyceridaemia together with a high prevalence of diabetes might explain why REDUCE-IT^9^ showed that lowering triglycerides reduced CVD risk. However, why the STRENGTH^10^ trial did not achieve its primary endpoint is not clear. It could be due to low treatment adherence^44^ and other reasons including chance.

Our study might provide some guidance for current and future clinical trials investigating the effect of lowering triglycerides on CVD. Presently, at least 29 current and future trials registered on the ClinicalTrials.gov website are designed to investigate the protective effect against CVD by triglyceride-lowering therapies via omega-3 fatty acid (see Supplementary material online, *Table S1^3^*), niacin (see Supplementary material online, *Table S1^4^*), or fibrate (see Supplementary material online, *Table S1^5^*). Among these 29 trials, 9 have a status of active but not recruiting, 15 recruiting, and 5 not yet recruiting (see Supplementary material online, *Tables S13–S15*). However, only one of the 29 trials uses high triglycerides as an inclusion criterion (NCT04562467, Supplementary material online, *Table S1^3^*) and none of them use both diabetes and high triglycerides as inclusion criteria.

The current study seemed inconsistent with the REDUCE-IT, as the latter showed that the triglyceride-lowering drug icosapent ethyl reduced CVD events in both diabetic and non-diabetic patients.^9^ However, the exact mechanisms underlying the CVD-lowering effect of icosapent ethyl are unclear, and it is possible that lowering triglycerides might not be the main mechanism. This speculation seemed to be supported by the following observation: (i) baseline triglyceride levels (≥150 vs. <150 mg/dL or ≥200 vs. <200 mg/dL) had no influence on the CVD-lowering effect of icosapent ethyl; and (ii) the attainment of triglyceride levels of ≥150 vs. <150 mg/dL at 1 year after randomization had no influence on the efficacy of icosapent ethyl.^9^ The REDUCE-IT investigators^9^ suggested that other mechanisms may contribute to the observed beneficial effect of icosapent ethyl and the proposed mechanisms included antiplatelet effect, stabilization or regression of coronary plaque, and anti-inflammatory effect associated with icosapent ethyl.

### 4.1 Strengths and limitations

This study has similar strengths to those previously reported.^18,20,22^ In brief, strengths include a large sample size (*n* = 26 570), a prospective study design, the use of a nationally representative sample of US adults, and adjustment for a large number of confounding factors. This study also has a number of limitations as previously reported^18,20,22^: (i) triglycerides were only measured at one time point, which may result in misclassification. Nevertheless, such misclassification would tend to result in an underestimate rather than an overestimate of risk due to the effect of regression dilution bias; (ii) mortality outcomes were ascertained by linkage to the NDI records with a probabilistic match, which may lead to misclassification. However, a prior validation study showed that the matching method had high accuracy (98.5%).^45^ In addition, this study does not represent the whole US population. The Hispanic subcohort counted for 27.7% of the whole cohort, which is higher than the percentage of the Hispanic subpopulation in the USA (18.7% in 2020). This difference was explained by the NHANES design: the NHANES cycles from 1988 to 2014 were designed to oversample the Hispanic subpopulation. This oversampling aimed to obtain sufficient numbers of Hispanic persons and to increase the reliability and precision of estimates of health status indicators for this subpopulation. Therefore, the results of the current study may not be extrapolated to the entire US population.

## 5. Conclusion

This study demonstrated that higher fasting triglycerides were associated with a higher risk of CVD mortality in people with diabetes but not in those without diabetes. Triglycerides may be a therapeutic target for lowering CVD mortality in people with both diabetes and hypertriglyceridaemia. A diabetes-induced sensitization to hypertriglyceridaemia hypothesis was proposed to describe the association between hypertriglyceridaemia and CVD mortality (*Figure 1*).

## Supplementary material

Supplementary material is available at *Cardiovascular Research* online.

## Authors’ contributions

Conceptualization: Y.W., Y.F.; data analysis: Y.W.; writing—original draft preparation, Y.W., Y.F.; writing—review and editing: Y.W., Y.F., D.J.M., F.J.C., C.G.S., G.R.D., J.G.; funding acquisition, Y.W.

## Supplementary Material

cvac124_Supplementary_Data

## Data Availability

All data in the current analysis are publicly available on the NHANES website.
