# Supplementary material for: Fasting triglycerides are positively associated with cardiovascular mortality risk in people with diabetes
Source: Cardiovasc Res. 2022 Jul 29;119(3):826–34. doi: 10.1093/cvr/cvac124 (PMC10153411; doi:10.1093/cvr/cvac124)
Supplement: cvac124_Supplementary_Data [file cvac124_supplementary_data.pdf]

Supplemental Material

Fasting triglycerides are positively associated with cardiovascular mortality risk in people with diabetes

Short title: Triglycerides and CVD mortality

Authors: Yutang Wang,[1,#] Yan Fang,[1] Dianna J. Magliano,[2] Fadi J. Charchar,[1] Christopher G. Sobey,[3] Grant R. Drummond,[3] Jonathan Golledge[4,5]

[1]Discipline of Life Science, School of Science, Psychology and Sport, Federation University Australia, Ballarat, VIC, 3350, Australia.

[2]Diabetes and Population Health, Baker Heart and Diabetes Institute, Melbourne, VIC, Australia

[3]Centre for Cardiovascular Biology and Disease Research and Department of Microbiology, Anatomy, Physiology & Pharmacology, School of Agriculture, Biomedicine & Environment, La Trobe University, Melbourne, VIC, Australia

[4]Queensland Research Centre for Peripheral Vascular Disease, College of Medicine and Dentistry, James Cook University, Townsville, QLD, Australia

[5]Department of Vascular and Endovascular Surgery, The Townsville University Hospital, Townsville, QLD, Australia

Correspondence to: Yutang Wang, Discipline of Life Science, School of Science, Psychology and Sport, Federation University Australia, University Drive, Mt Helen, VIC, 3350, Australia. Tel: 61-3-53279394; E-mail: yutang.wang@federation.edu.au

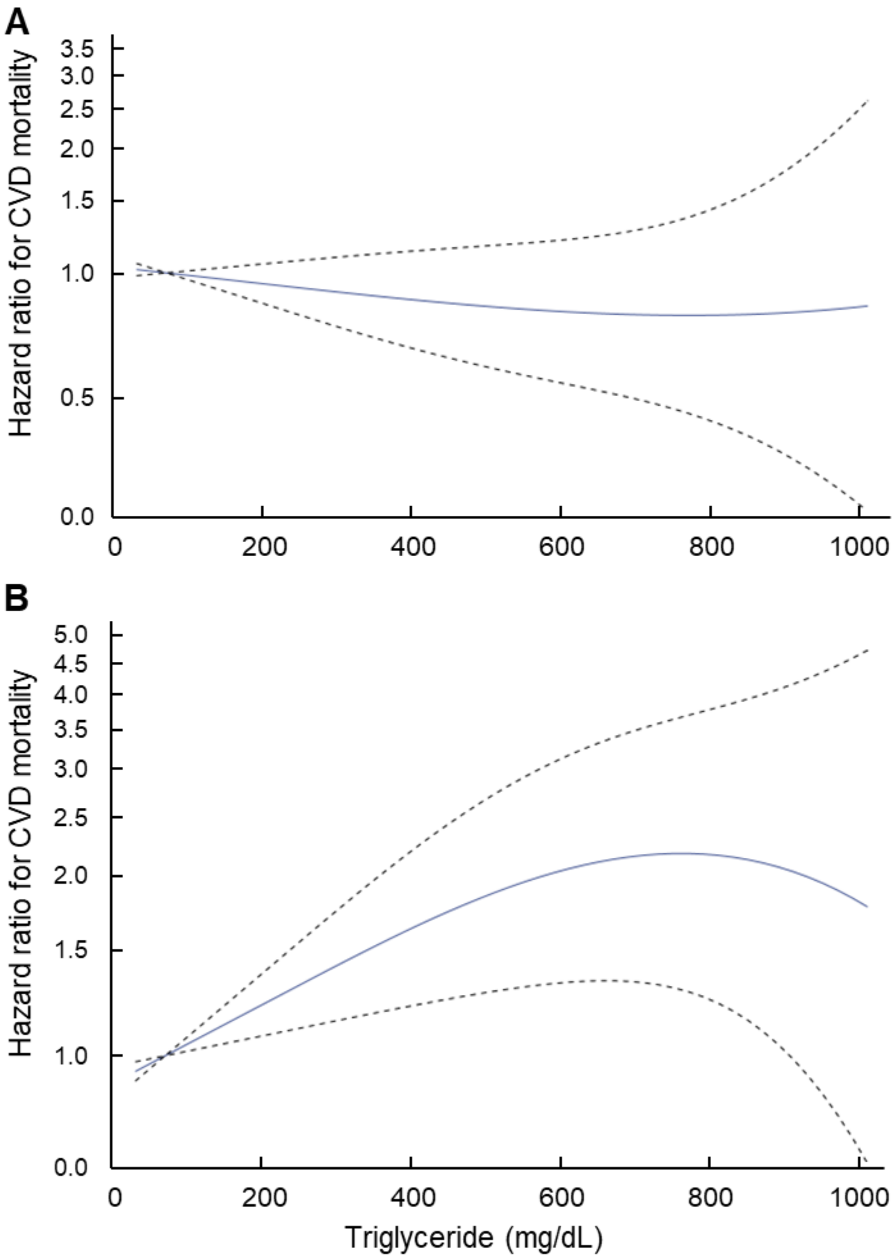

Supplemental Figure 1. Adjusted restricted cubic spline plots of triglycerides for CVD mortality risks. **A**, Participants without diabetes. **B**, Participants with diabetes (*P*=0.011, for the non-linearity test). The analysis was adjusted for age, sex, ethnicity, obesity, poverty-income ratio, education, physical activity, alcohol consumption, smoking status, survey period, hypercholesterolemia, hypertension, family history of diabetes, duration of diabetes, and diabetes medications. CVD, cardiovascular disease.

Supplemental Table 1. Recent 13 randomized controlled trials (RCTs) since 2010 on triglyceride-lowering therapies with cardiovascular outcomes as primary endpoints

Ref	Trial names	Publication year	Patient population	Intervention	Primary endpoint	Was primary endpoint achieved?
Omega-3 trials						
1	Omega	2010	3,851 patients with recent MI (3 to 14 days)	EPA + DHA vs. placebo	sudden cardiac death	No
2	Alpha Omega	2010	4,837 patients with MI	EPA +DHA, or alpha-linolenic acid vs. placebo	fatal and nonfatal CVD events or cardiac interventions	No
3	SU.FOL.OM3	2010	2,501 patients with MI, unstable angina, or ischaemic stroke	EPA + DHA vs. placebo	nonfatal MI, stroke, or CVD death	No
4	ORIGIN	2012	12,536 patients with dysglycemia and high CVD risk	EPA + DHA vs. placebo	CVD death	No
5	Risk and Prevention	2013	12,513 patients with multiple CVD risk factors	EPA + DHA vs. placebo	nonfatal MI or nonfatal stroke	No
6	ASCEND	2018	15,480 patients with diabetes but without atherosclerotic CVD	EPA + DHA vs. placebo	nonfatal MI, stroke, transient ischemic attack, or vascular death, excluding confirmed intracranial hemorrhage	No
7	VITAL	2019	25,871 participants from a general population	EPA + DHA vs. placebo	major CVD events (MI, stroke, or CVD death), or invasive cancer	No
8	REDUCE-IT	2019	8,179 patients with hypertriglyceridemia (fasting triglyceride 135 to 499 mg/dL) Diabetes: 58.5%	icosapent ethyl vs. placebo	CVD death, nonfatal MI, nonfatal stroke, coronary revascularization, or unstable angina	Yes
9	STRENGTH	2020	13,078 patients with high CVD risk	EPA + DHA vs. placebo	CVD death, MI, stroke, coronary	No

					revascularization, or unstable angina requiring hospitalization	
10	OMEMI	2021	1,027 elderly patients with recent MI (2-8 weeks)	EPA + DHA vs. placebo	nonfatal MI, unscheduled revascularization, stroke, all-cause death, or heart failure hospitalization after 2 years	No
Niacin trials						
11	AIM-HIGH	2011	3,414 patients with prior CVD	niacin vs. placebo	CHD death, nonfatal MI, or ischemic stroke, etc	No
12	HPS2-THRIVE	2014	25,673 patients with prior CVD	niacin + laropiprant vs. placebo	nonfatal MI, cardiac death, stroke,<br>or arterial revascularization	No
Fibrate trials						
13	ACCORD-Lipid	2011	5,518 patients with type 2 diabetes	fenofibrate vs. placebo	fatal and nonfatal CHD, or stroke	No

ACCORD, Action to Control Cardiovascular Risk in Diabetes; AIM-HIGH, Atherothrombosis Intervention in Metabolic Syndrome with Low HDL/High Triglycerides, Impact on Global Health Outcomes; Alpha Omega, Study of Omega-3 Fatty Acids and Coronary Mortality; ASCEND, A Study of Cardiovascular Events in Diabetes; BIP, Bezafibrate Infarction Prevention; CHD, coronary heart disease; CVD, cardiovascular disease; DHA, docosahexaenoic acid; EPA, eicosapentaenoic acid; FIELD, Fenofibrate Intervention and Event Lowering in Diabetes; HDL, high-density lipoprotein; HPS2-THRIVE, Heart Protection Study 2–Treatment of HDL to Reduce the Incidence of Vascular Events; MI, myocardial infarction; OMEGA, Effect of Omega 3-Fatty Acids on the Reduction of Sudden Cardiac Death After Myocardial Infarction; OMEMI, Omega-3 Fatty Acids in Elderly Patients With Myocardial Infarction; ORIGIN, Outcome Reduction with an Initial Glargine Intervention; ref, reference; REDUCE-IT, Reduction of Cardiovascular Events with Icosapent Ethyl–Intervention Trial; STRENGTH, Long-Term Outcomes Study to Assess Statin Residual Risk with Epanova in High Cardiovascular Risk Patients with Hypertriglyceridemia; SU.FOL.OM3, Supplémentation en Folates et Omega-3; VITAL, Vitamin D and Omega A3 Trial; vs., versus.

References

1. Rauch B, Schiele R, Schneider S, Diller F, Victor N, Gohlke H, Gottwik M, Steinbeck G, Del Castillo U, Sack R, et al. OMEGA, a randomized, placebo-controlled trial to test the effect of highly purified omega-3 fatty acids on top of modern guideline-adjusted therapy after myocardial infarction. Circulation. 2010;122:2152-9.
2. Kromhout D, Giltay EJ and Geleijnse JM. n–3 Fatty Acids and Cardiovascular Events after Myocardial Infarction. New England Journal of Medicine. 2010;363:2015-2026.
3. Galan P, Kesse-Guyot E, Czernichow S, Briancon S, Blacher J and Hercberg S. Effects of B vitamins and omega 3 fatty acids on cardiovascular diseases: a randomised placebo controlled trial. BMJ. 2010;341:c6273.
4. The ORIGIN Trial Investigators. n–3 Fatty Acids and Cardiovascular Outcomes in Patients with Dysglycemia. New England Journal of Medicine. 2012;367:309-318.

5. Roncaglioni MC, Tombesi M, Avanzini F, Barlera S, Caimi V, Longoni P, Marzona I, Milani V, Silletta MG, Tognoni G, et al. n-3 fatty acids in patients with multiple cardiovascular risk factors. N Engl J Med. 2013;368:1800-8.
6. The ASCEND Study Collaborative Group. Effects of n−3 Fatty Acid Supplements in Diabetes Mellitus. New England Journal of Medicine. 2018;379:1540-1550.
7. Manson JE, Cook NR, Lee IM, Christen W, Bassuk SS, Mora S, Gibson H, Albert CM, Gordon D, Copeland T, et al. Marine n-3 Fatty Acids and Prevention of Cardiovascular Disease and Cancer. N Engl J Med. 2019;380:23-32.
8. Bhatt DL, Steg PG, Miller M, Brinton EA, Jacobson TA, Ketchum SB, Doyle RT, Jr., Juliano RA, Jiao L, Granowitz C, et al. Cardiovascular Risk Reduction with Icosapent Ethyl for Hypertriglyceridemia. N Engl J Med. 2019;380:11-22.
9. Nicholls SJ, Lincoff AM, Garcia M, Bash D, Ballantyne CM, Barter PJ, Davidson MH, Kastelein JJP, Koenig W, McGuire DK, et al. Effect of High-Dose Omega-3 Fatty Acids vs Corn Oil on Major Adverse Cardiovascular Events in Patients at High Cardiovascular Risk: The STRENGTH Randomized Clinical Trial. Jama. 2020;324:2268-2280.
10. Kalstad AA, Myhre PL, Laake K, Tveit SH, Schmidt EB, Smith P, Nilsen DWT, Tveit A, Fagerland MW, Solheim S, et al. Effects of n-3 Fatty Acid Supplements in Elderly Patients After Myocardial Infarction. Circulation. 2021;143:528-539.
11. The AIM-HIGH Investigators. Niacin in Patients with Low HDL Cholesterol Levels Receiving Intensive Statin Therapy. New England Journal of Medicine. 2011;365:2255-2267.
12. The HPS2-THRIVE Collaborative Group. Effects of Extended-Release Niacin with Laropiprant in High-Risk Patients. New England Journal of Medicine. 2014;371:203-212.
13. Elam M, Lovato L and Ginsberg H. The ACCORD-Lipid study: implications for treatment of dyslipidemia in Type 2 diabetes mellitus. Clin Lipidol. 2011;6:9-20

Supplemental Table 2. Interaction of diabetes with triglyceride for CVD mortality risks

	HR *	95% CI	*P* value
LnTG X diabetes †	1.30	1.05-1.61	0.015

Abbreviations: CI, confidence interval; HR, hazard ratio; LnTG, natural log-transformed triglycerides.

* Adjusted for age, sex, ethnicity, obesity, poverty-income ratio, education, physical activity, alcohol consumption, smoking status, survey period, hypercholesterolemia, hypertension, family history of diabetes, duration of diabetes, diabetes medications, diabetes, and natural log-transformed triglycerides (LnTG).

† The interaction factor, computed as natural log-transformed triglycerides multiplied by diabetes (yes and no, coded as 1 and 0, respectively). The resulting interaction factor was treated as a continuous variable in the interaction analysis.

Supplemental Table 3. Triglyceride (natural log-transformed) and risk for CVD mortality among 26,570 adults, stratified by LDL cholesterol

LDL cholesterol (mg/dL)	N	HR *	95% CI	*P* value
Participants without diabetes				
≤ 55	368	0.70	0.14-3.61	0.668
55.1-70	882	0.92	0.31-2.70	0.878
70.1-100	4,769	0.82	0.57-1.18	0.293
>100	13,822	1.01	0.84-1.21	0.932
Unknown (missing data)	2,751	0.91	0.71-1.16	0.438
Participants with diabetes				
≤ 55	139	234	0-2.6E+15	0.722
55.1-70	301	1.00	0.32-3.18	1.000
70.1-100	946	2.06	1.02-4.15	0.044
>100	2,165	1.16	0.83-1.63	0.375
Unknown (missing data)	427	1.27	0.92-1.76	0.144

Abbreviations: CI, confidence interval; CVD, cardiovascular disease; HR, hazard ratio; LDL, low-density lipoprotein.

* Adjusted for age, sex, ethnicity, obesity, poverty-income ratio, education, physical activity, alcohol consumption, smoking status, survey period, hypercholesterolemia, hypertension, family history of diabetes, duration of diabetes, and diabetes medications.

Supplemental Table 4. Sensitivity analysis of the association of LnTG with CVD mortality risk when diabetes was defined by era-specific fasting glucose levels *

Models	HR	95% CI	*P* value
All participants (N=26,515)			
Model 1	1.23	1.12-1.36	<0.001
Model 2	1.16	1.04-1.28	0.006
Model 3	1.12	1.01-1.24	0.037
Model 4	1.05	0.94-1.16	0.395
Participants without diabetes (N=24,176)			
Model 1	1.08	0.96-1.20	0.205
Model 2	0.99	0.88-1.12	0.907
Model 3	0.96	0.85-1.08	0.478
Model 4	0.95	0.84-1.07	0.368
Participants with diabetes (N=2,339)			
Model 1	1.55	1.25-1.92	<0.001
Model 2	1.41	1.13-1.77	0.003
Model 3	1.38	1.10-1.72	0.006
Model 4	1.41	1.12-1.77	0.003

Abbreviations: CI, confidence interval; CVD, cardiovascular disease; HR, hazard ratio; LnTG, natural log-transformed triglycerides.

* In this sensitivity analysis, diabetes was defined as glucose ≥140 mg/dL for participants from NHANES III (1988-1994) or ≥126 mg/dL for participants from NHANES 1999-2014, due to the change of diabetes diagnostic criteria by the American Diabetes Association in 1997. Fifty-five participants were excluded due to lacking glucose data and 26,515 participants were included in the sensitivity analysis.

Model 1: adjusted for age, sex, and ethnicity; Model 2: adjusted for age, sex, ethnicity, obesity, poverty-income ratio, education, physical activity, alcohol consumption, smoking status, and survey period; Model 3: adjusted for all the factors in Model 2 plus hypercholesterolemia and hypertension; Model 4: adjusted for all the factors in Model 3 plus diabetes, family history of diabetes, duration of diabetes, and diabetes medications.

Supplemental Table 5. Sensitivity analysis of the association of LnTG with CVD mortality risk when diabetes was defined as fasting glucose levels ≥ 126 mg/dL [*]

Models	HR	95% CI	*P* value
All participants (N=26,515)			
Model 1	1.24	1.12-1.36	<0.001
Model 2	1.16	1.05-1.28	0.005
Model 3	1.12	1.01-1.24	0.033
Model 4	1.05	0.94-1.16	0.414
Participants without diabetes (N=23,986)			
Model 1	1.04	0.93-1.17	0.476
Model 2	0.96	0.86-1.09	0.549
Model 3	0.93	0.83-1.05	0.254
Model 4	0.93	0.82-1.05	0.216
Participants with diabetes (N=2,529)			
Model 1	1.55	1.26-1.89	<0.001
Model 2	1.44	1.16-1.77	0.001
Model 3	1.40	1.13-1.73	0.002
Model 4	1.43	1.16-1.78	0.001

Abbreviations: CI, confidence interval; CVD, cardiovascular disease; HR, hazard ratio; LnTG, natural log-transformed triglyceride

[*] In this sensitivity analysis, diabetes was defined as fasting plasma glucose ≥126 mg/dL for all participants. Fifty-five participants were excluded due to lacking glucose data and 26,515 participants were included in the sensitivity analysis.

Model 1: adjusted for age, sex, and ethnicity; Model 2: adjusted for age, sex, ethnicity, obesity, poverty-income ratio, education, physical activity, alcohol consumption, smoking status, and survey period; Model 3: adjusted for all the factors in Model 2 plus hypercholesterolemia and hypertension; Model 4: adjusted for all the factors in Model 3 plus diabetes, family history of diabetes, duration of diabetes, and diabetes medications.

Supplemental Table 6. Sensitivity analysis of the association of LnTG with CVD mortality risk among 26,570 adults after further adjustment for the use of lipid-lowering medications

Models	HR	95% CI	*P* value
All participants (N=26,570)			
Model 1	1.06	0.95-1.17	0.315
Model 2	1.06	0.95-1.17	0.296
Participants without diabetes (N=22,592)			
Model 1	0.95	0.83-1.07	0.382
Model 2	0.95	0.83-1.08	0.404
Participants with diabetes (N=3,978)			
Model 1	1.30	1.08-1.56	0.006
Model 2	1.30	1.08-1.57	0.006

Abbreviations: CI, confidence interval; CVD, cardiovascular disease; HR, hazard ratio; LnTG, natural log-transformed triglycerides.

Model 1: adjusted for age, sex, ethnicity, obesity, poverty-income ratio, education, physical activity, alcohol consumption, smoking status, survey period, hypercholesterolemia, hypertension, diabetes, family history of diabetes, duration of diabetes, and diabetes medications.

Model 2: adjusted for all the factors in Model 1 plus the use of lipid-lowering medications (yes, no, or unknown).

Supplemental Table 7. Sensitivity analysis of the association of LnTG with CVD mortality risk among 23,496 adults after exclusion of those using lipid-lowering medications *

Models	HR	95% CI	*P* value
All participants (N=23,496)			
Model 1	1.19	1.07-1.33	0.001
Model 2	1.12	1.00-1.25	0.049
Model 3	1.09	0.98-1.22	0.116
Model 4	1.04	0.93-1.16	0.522
Participants without diabetes (N=20,758)			
Model 1	1.02	0.90-1.16	0.709
Model 2	0.95	0.84-1.09	0.491
Model 3	0.94	0.82-1.07	0.337
Model 4	0.94	0.82-1.08	0.359
Participants with diabetes (N=2,738)			
Model 1	1.38	1.12-1.68	0.002
Model 2	1.28	1.04-1.58	0.020
Model 3	1.26	1.02-1.56	0.033
Model 4	1.32	1.06-1.64	0.013

Abbreviations: CI, confidence interval; CVD, cardiovascular disease; HR, hazard ratio; LnTG, natural log-transformed triglycerides.

* A total of 3,074 participants took lipid-lowering medications. After exclusion of those participants, 23,496 participants were included in the sensitivity analysis.

Model 1: adjusted for age, sex, and ethnicity; Model 2: adjusted for age, sex, ethnicity, obesity, poverty-income ratio, education, physical activity, alcohol consumption, smoking status, and survey period; Model 3: adjusted for all the factors in Model 2 plus hypercholesterolemia and hypertension; Model 4: adjusted for all the factors in Model 3 plus diabetes, family history of diabetes, duration of diabetes, and diabetes medications.

Supplemental Table 8. Sensitivity analysis of the association of LnTG with risk for CVD mortality among 25,956 adults* when the analysis was adjusted for systolic blood pressure (continuous) rather than hypertension (categorical)

	HR†	95% CI	*P* value
All participants (N=26,570)	1.03	0.93-1.14	0.594
Participants without diabetes (N=22,592)	0.91	0.80-1.04	0.168
Participants with diabetes (N=3,978)	1.31	1.09-1.58	0.005

Abbreviations: CI, confidence interval; CVD, cardiovascular disease; HR, hazard ratio; LnTG, natural log-transformed triglycerides.

* A total of 614 participants did not have systolic blood pressure data. After exclusion of those participants, the remaining 25,956 participants were included in the sensitivity analysis.

† Adjusted for age, sex, ethnicity, obesity, poverty-income ratio, education, physical activity, alcohol consumption, smoking status, survey period, hypercholesterolemia, systolic blood pressure (natural log-transformed), diabetes, family history of diabetes, duration of diabetes, and diabetes medications.

Supplemental Table 9. Sensitivity analysis of the association of LnTG with risk for CVD mortality among 26,569 adults* when the analysis was adjusted for total cholesterol (continuous) rather than hypercholesterolemia (categorical)

Models	HR	95% CI	*P* value
All participants (N=26,569)			
Model 1	1.06	0.95-1.17	0.315
Model 2	1.04	0.93-1.16	0.520
Participants without diabetes (N=22,591)			
Model 1	0.95	0.83-1.07	0.381
Model 2	0.94	0.82-1.08	0.390
Participants with diabetes (N=3,978)			
Model 1	1.30	1.08-1.56	0.006
Model 2	1.24	1.02-1.52	0.033

Abbreviations: CI, confidence interval; CVD, cardiovascular disease; HR, hazard ratio; LnTG, natural log-transformed triglycerides.

* One participant did not have total cholesterol data. After exclusion of that participant, the remaining 26,569 participants were included in the sensitivity analysis.

Model 1: adjusted for age, sex, ethnicity, obesity, poverty-income ratio, education, physical activity, alcohol consumption, smoking status, survey period, hypertension, diabetes, family history of diabetes, duration of diabetes, diabetes medications, and hypercholesterolemia.

Model 2: adjusted for all the factors in Model 1 except that hypercholesterolemia was replaced by total cholesterol (natural log-transformed).

Supplemental Table 10. Sensitivity analysis of the association of LnTG with risk for CVD mortality among 26,527 adults* when the analysis was adjusted for HDL-cholesterol (continuous) rather than hypercholesterolemia (categorical)

Models	HR	95% CI	*P* value
All participants (N=26,527)			
Model 1	1.06	0.95-1.18	0.298
Model 2	1.06	0.94-1.19	0.341
Participants without diabetes (N=22,564)			
Model 1	0.95	0.84-1.08	0.425
Model 2	0.95	0.83-1.10	0.483
Participants with diabetes (N=3,963)			
Model 1	1.30	1.08-1.58	0.006
Model 2	1.32	1.07-1.63	0.011

Abbreviations: CI, confidence interval; CVD, cardiovascular disease; HDL, high-density lipoprotein; HR, hazard ratio; LnTG, natural log-transformed triglycerides.

*A total of 43 participants did not have HDL cholesterol data. After exclusion of these participants, the remaining 26,527 participants were included in the sensitivity analysis.

Model 1: adjusted for age, sex, ethnicity, obesity, poverty-income ratio, education, physical activity, alcohol consumption, smoking status, survey period, hypertension, diabetes, family history of diabetes, duration of diabetes, diabetes medications, and hypercholesterolemia.

Model 2: adjusted for all the factors in Model 1 except that hypercholesterolemia was replaced by HDL cholesterol (natural log-transformed).

Supplemental Table 11. Sensitivity analysis of the association of LnTG with risk for CVD mortality among 26,526 adults* when the analysis was adjusted for non-HDL cholesterol (continuous) rather than hypercholesterolemia (categorical)

Models	HR	95% CI	*P* value
All participants (N=26,526)			
Model 1	1.06	0.95-1.18	0.299
Model 2	1.04	0.92-1.17	0.530
Participants without diabetes (N=22,563)			
Model 1	0.95	0.84-1.08	0.425
Model 2	0.95	0.83-1.10	0.493
Participants with diabetes (N=3,963)			
Model 1	1.30	1.08-1.58	0.006
Model 2	1.25	1.00-1.55	0.049

Abbreviations: CI, confidence interval; CVD, cardiovascular disease; HDL, high-density lipoprotein; HR, hazard ratio; LnTG, natural log-transformed triglycerides.

*A total of 44 participants did not have non-HDL cholesterol data. After exclusion of these 44 participants, the remaining 26,526 participants were included in the sensitivity analysis.

Model 1: adjusted for age, sex, ethnicity, obesity, poverty-income ratio, education, physical activity, alcohol consumption, smoking status, survey period, hypertension, diabetes, family history of diabetes, duration of diabetes, diabetes medications, and hypercholesterolemia.

Model 2: adjusted for all the factors in Model 1 except that hypercholesterolemia was replaced by non-HDL cholesterol (natural log-transformed).

Supplemental Table 12. Diabetes prevalence and triglyceride requirement in the recent 13 randomized controlled trials investigating the effect of triglyceride-lowering therapies on CVD

Reference	Trial names	Diabetes prevalence	Is high triglyceride an inclusion criterion?
Omega-3 trials			
1	Omega	27%	No
2	Alpha Omega	21%	No
3	SU.FOL.OM3	unknown	No
4	ORIGIN	<50%	No
5	Risk and Prevention	60%	No
6	ASCEND	100%	No, triglyceride was not measured
7	VITAL	13.7%	No
8	REDUCE-IT	58.5%	Yes, 135-499 mg/dL
9	STRENGTH	70%	Yes, 180-500 mg/dL
10	OMEMI	20.7%	No
Niacin trials			
11	AIM-HIGH	33.9%	Yes, 150-400 mg/dL
12	HPS2-THRIVE	32.3%	No
Fibrate trials			
13	ACCORD-Lipid	100%	No

See Supplemental Table 1 for abbreviations and the reference for each trial.

Supplemental Table 13. Current and future trials on omega-3 and cardiovascular disease (CVD)

No.	Trial identifier	Trial name	Trial status	Is diabetes an inclusion criterion?	Is high triglyceride an inclusion criterion?	Intervention
1	NCT01343342	Genes, Omega-3 Fatty Acids and Cardiovascular Disease Risk Factors (FAS)	Active, not recruiting	No	No	n-3 PUFA supplementation
2	NCT02178410	VITAL Rhythm Study	Active, not recruiting	No	No	Omega-3 fatty acids (fish oil) or vitamin D3
3	NCT01630213	Impact of Vitamin D Supplementation on Cardiac Structure and Function	Active, not recruiting	No	No	Vitamin D3 + fish oil
4	NCT01785004	Vitamin D and Omega-3 Adiposity Trial (VITAL Adiposity)	Active, not recruiting	No	No	Omega-3 fatty acids (fish oil) or vitamin D3
5	NCT01653678	Vitamin D and Omega-3 Hypertension Trial (VITAL Hypertension)	Active, not recruiting	No	No	Omega-3 fatty acids (fish oil) or vitamin D3
6	NCT02757872	Effects of Vitamin D and Fish Oil on the Kidney in Hypertensives	Active, not recruiting	No, diabetes was excluded	No	Omega-3 fatty acids (fish oil) or vitamin D3
7	NCT03192410	Prospective Cohort Study of 4,837 Post-myocardial Infarction Patients (Alpha Omega Cohort)	Active, not recruiting	No	No	Dietary intake
8	NCT01953705	n-3 PUFA for Vascular Cognitive Aging	Active, not recruiting	No	No	Omega-3 PUFA
9	NCT04658433	The Effect of Omega -3 Supplements on the Serum Levels of ACE/ACE2 Ratio as a Potential Key in Cardiovascular Disease and COVID-19; A Randomized Clinical Trial in the	Recruiting	No	No	Omega-3 fatty acids

		Covid-19 Uninfected Jordanian People				
10	NCT04763291	Cardiovascular and InflammAging Study	Recruiting	No	No	Juice Plus+ Omega blend
11	NCT04031508	Effect of a Parenteral Emulsion With Omega3 on PPHN	Recruiting	No	No	Lipid injectable emulsion with fish oil
12	NCT04562467	The Use of Icosapent Ethyl on Vascular Progenitor Cells in Individuals With Elevated Cardiovascular Risk	Recruiting	Partially	Yes	Icosapent Ethyl 1000 mg oral capsule
13	NCT03576989	Impact of Omega-3 Fatty Acid Oral Therapy on Healing of Chronic Venous Leg Ulcers in Older Adults	Recruiting	No	No	EPA+DHA
14	NCT04499820	Effect of OMEGA3 Supplementation in Diabetic Retinopathy	Recruiting	Yes	No	Omega-3 supplementation
15	NCT04632407	Can Flaxseed Prevent Broken Hearts in Women With Breast Cancer Study?	Recruiting	No	No	Flax "milk"
16	NCT04120077	Combined Effects of Diabetes Self-Management Education and Nutritional Supplementation on Visual Function and Retinopathy	Recruiting	Partially, also includes prediabetes	No	Multi-component formula or omega-3 fatty acid supplement
17	NCT04496817	Enriched Eggs for Retina Health in Type 2 Diabetes	Not yet recruiting	Yes	No	Docosahexaenoic acid and lutein enriched eggs vs regular eggs
18	NCT04386525	Omega 3 and Ischemic Stroke; Fish Oil as an Option (OmegaStroke)	Not yet recruiting	No	No	Omega-3 fish oil

Abbreviations: DHA, docosahexaenoic acid; EPA, eicosapentaenoic acid; PUFA, polyunsaturated fatty acid.

Supplemental Table 14. Current and future trials on niacin and cardiovascular disease (CVD)

No.	Trial identifier	Trial name	Trial status	Is diabetes an inclusion criterion?	Is high triglyceride an inclusion criterion?	Intervention
1	NCT04870866	NAD Supplementation to Prevent Progressive Neurological Disease in Ataxia Telangiectasia	Active, not recruiting	No	No	Nicotinamide ribonucleoside
2	NCT04040959	Nicotinamide Riboside Supplementation for Treating Arterial Stiffness and Elevated Systolic Blood Pressure in Patients With Moderate to Severe CKD	Recruiting	No	No	Nicotinamide riboside
3	NCT03821623	Nicotinamide Riboside for Treating Elevated Systolic Blood Pressure and Arterial Stiffness in Middle-aged and Older Adults	Recruiting	No, diabetes was excluded	No	Nicotinamide riboside
4	NCT04528004	Mechanistic Studies of Nicotinamide Riboside in Human Heart Failure	Recruiting	No	No	Nicotinamide riboside
5	NCT04112043	Nicotinamide Riboside as an Enhancer of Exercise Therapy in Hypertensive Older Adults (The NEET Trial)	Recruiting	No	No	Nicotinamide Riboside
6	NCT04913805	Matching Perfusion and Metabolic Activity in HFpEF	Recruiting	No	No	Potassium nitrate + propionyl-L-Carnitine + nicotinamide riboside
7	NCT03743636	Nicotinamide Riboside With and Without Resveratrol to Improve Functioning in Peripheral Artery Disease	Recruiting	No	No	Nicotinamide riboside

8	NCT04750616	NAD+ Augmentation in Cardiac Surgery Associated Myocardial Injury Trial	Recruiting	No	No	Niacinamide
9	NCT04632121	Oral Nicorandil in ST Elevation Myocardial Infarction Patients Undergoing Primary Percutaneous Coronary Intervention	Not yet recruiting	No	No	Nicorandil
10	NCT04903210	Nicotinamide Mononucleotide in Hypertensive Patients	Not yet recruiting	No	No	Nicotinamide mononucleotide

Abbreviations: CKD, chronic kidney disease; NAD, nicotinamide adenine dinucleotide.

Supplemental Table 15. Current and future trials on fibrate and cardiovascular disease (CVD)

No.	Trial identifier	Trial name	Trial status	Is diabetes an inclusion criterion?	Is high triglyceride an inclusion criterion?	Intervention
1	NCT04140201	Effect of Lipid Lowering Agents on Diabetic Retinopathy and Cardiovascular Risk of Diabetic Patients	Not yet recruiting	Yes	No	Simvastatin, or fenofibrate, or omega-3 fatty acid
